# Traditional therapies of Zhuang medicine improve pain and joint dysfunction of patients in rheumatoid arthritis: A protocol for systematic review and meta-analysis

**DOI:** 10.1097/MD.0000000000022264

**Published:** 2020-09-25

**Authors:** Yehao Luo, Donghan Xu, Zhiyong Cao, Qiuxia Chen, Lizhen Wang, Gang Fang, Yuzhou Pang

**Affiliations:** a Guangxi University of Traditional Chinese Medicine, Nanning, Guangxi Province; b Macau University of Science and Technology, Macau; c Hubei Minzu University, Wuhan; d Hospital of Chengdu University of Traditional Chinese Medicine, Chengdu, Sichuan Province; e Guangxi Zhuang Yao Medicine Center of Engineering and Technology, Guangxi University of Chinese Medicine, Nanning, Guangxi Province, China.

**Keywords:** joint dysfunction, pain, protocol, rheumatoid arthritis, systematic review, Zhuang medicine

## Abstract

**Background:**

Rheumatoid arthritis (RA) is a common chronic inflammatory autoimmune disease, which can lead to joint destruction, dysfunction, finally deformity. Currently, Western medicine treats it with disease-modifying antireheumatic drugs, NSAIDs, glucocorticoid, biological agents, etc, which can induce adverse drug reactions. And now, as an important mean of treating RA, Zhuang medicine has been widely used in clinics, and has achieved significant efficacy.

**Methods and analysis:**

The following databases will be searched for relevant information before July 2020: PubMed, Embase, Cochrane Library, Web of Science, and China National Knowledge Infrastructure. Major results: levels of C-reactive protein, erythrocyte sedimentation rate, Rheumatoid factor. Secondary results: morning stiffness time, range of motion, arthralgia, joint tenderness index, joint swelling index, total effective rate, adverse event. Data will be collected independently by 2 researchers, and the risk of bias in meta analysis will be evaluated according to “Cochrane Handbook for Systematic Reviews of Interventions”. All data analysis will be conducted using Review Manager V.5.3. and Stata V.12.0.

**Results:**

The curative effect and safety of traditional therapies of Zhuang Medicine treatment for RA patients will be evaluated systematically.

**Conclusion:**

The systematic review of this study will summarize the currently published evidence of traditional therapies of Zhuang Medicine treatment for RA to further guide its promotion and application.

Open Science Framework (OSF) registration number: https://osf.io/c4xv3/.

## 1 Introduction

Rheumatoid arthritis (RA),as known to all, is a long-lasting disease that primarily affects the joints.^[1]^ Also, it is characterized by an aggressive inflammatory reaction of the small joints of the hand and foot, often accompanied by a positive serum rheumatoid factor, which can lead to joint deformity and dysfunction.^[2]^ However, the cause of RA is unclear, but genetic and environmental factors are involved.^[3]^ It is reported that the immune system affected by certain conditions could attack the joints. To the patients, RA has a significant negative impact on the ability to perform daily activities, including work and household tasks, and health-related quality of life, even it increases mortality. Globally, RA affects about 0.5 to 1 percent of adults in the developed countries, and it is more occupied in women at the middle age.^[4]^ Therefore, it is necessary to relieve the symptoms of RA, even cure. Modern Western Medicine mainly treats RA with traditional disease-modifying antireheumatic drugs (DMARDs), biologic agents, tofacitinib, glucocorticoids and treat-to-target approach.^[5]^ But partial patients unfortunately have to suffer from gastrointestinal adverse reactions which are induced by DMARDs, and a high price of the treatments that could add up the burden on the family.

Unexpectedly, we found that the treatment of traditional Chinese Medicine has a great impact on RA, not only relieve the pain and joints dysfunction but also raise the health-life quality. Have to commit, Zhuang medicine is one kind of treatments of traditional Chinese medicine. According to Zhuang medicine, RA belongs to range of “Fungcaep” (a unique term used in Zhuang medicine).^[6]^ The guideline for the treatments of RA is based on the theory of Zhuang medicine, moreover, the use of medicated thread moxibustion, cupping therapy with Zhuang medicine, exsanguainate therapy and other treatments with Zhuang medicine have been applied in clinics, which obtain an outstanding clinical efficiency.^[7]^ However, there is a lack of evidence of results of traditional therapies of Zhuang Medicine in treating RA. Therefore, the paper will evaluate the effectiveness and safety of traditional therapies of Zhuang medicine treatment for RA. This review will be the first evaluation of the impact of traditional therapies of Zhuang medicine treatment.

## 2 Objectives

In a randomized controlled trial (RCT), the efficacy and side effects of traditional therapies of Zhuang medicine in treating RA have been evaluated systematically. We expect to provide reference for RA treatment in the field of traditional Chinese medicine.

## 3 Methods

### 3.1 Study registration

The protocol of the systematic review has been registered.

Registration: OSF Preregisration.2020, Aug.19. osf.io/c4xv3/. This systematic review protocol will be conducted and reported strictly according to Preferred Reporting Items for Systematic Reviews and Meta-Analyses^[8]^ statement guidelines, and the important protocol amendments will be documented in the full review.

### 3.2 Inclusion and exclusion criteria for study selection

#### 3.2.1 Inclusion criteria

Inclusion criteria are all RCTs, which main treatment of RA is traditional therapies of Zhuang medicine. The language of the trials to be included only Chinese or English.

#### 3.2.2 exclusion criteria. Following studies will be excluded

1. Repeated publications
2. Review of literature and cases
3. Animal studies
4. Incomplete literature
5. Non-RCT

### 3.3 Types of participants

We will include RCTs of participants of 18 years or older, of any sex, race/ethnicity, and the patients are in accordance with diagnostic criteria of RA established by the American College of Rheumatology (ACR) in 1987 or the European alliance against Rheumatism (EULAR)/ACR in 2009 or the Chinese institute of Rheumatology in 2010 or the World Health Organization (WHO)in 2008. We will exclude advanced stage patients, severe joint deformities, and grade IV joint function; patients who are also participating in clinical trials for other drugs or treatments; patients with mental or legal disabilities should not be treated with this therapy; overlap other rheumatic diseases, such as systemic lupus erythematosus, Sjogren syndrome, severe knee osteoarthritis, etc; pregnant or lactating women, mental patients, etc; patients with severe diseases of the heart, brain, liver, kidney, or hematopoietic system.

### 3.4 Interventions and controls

Interventions included treatment with traditional therapies of Zhuang medicine. The control group only received conventional western medicine treatment. The routine treatment of each RCT may not be identical, but the use of traditional therapies of Zhuang medicine is the only difference between intervention and control.

### 3.5 Type of outcome measures

#### 3.5.1 Main outcomes

1. C-reactive protein;
2. erythrocyte sedimentation rate;
3. rheumatoid factor

#### 3.5.2 Additional outcomes

1. morning stiffness time;
2. range of motion;
3. arthralgia;
4. joint tenderness index;
5. joint swelling index;
6. total effective rate;
7. adverse events.

### 3.6 Search methods

#### 3.6.1 Search resources

**Figure 1:**
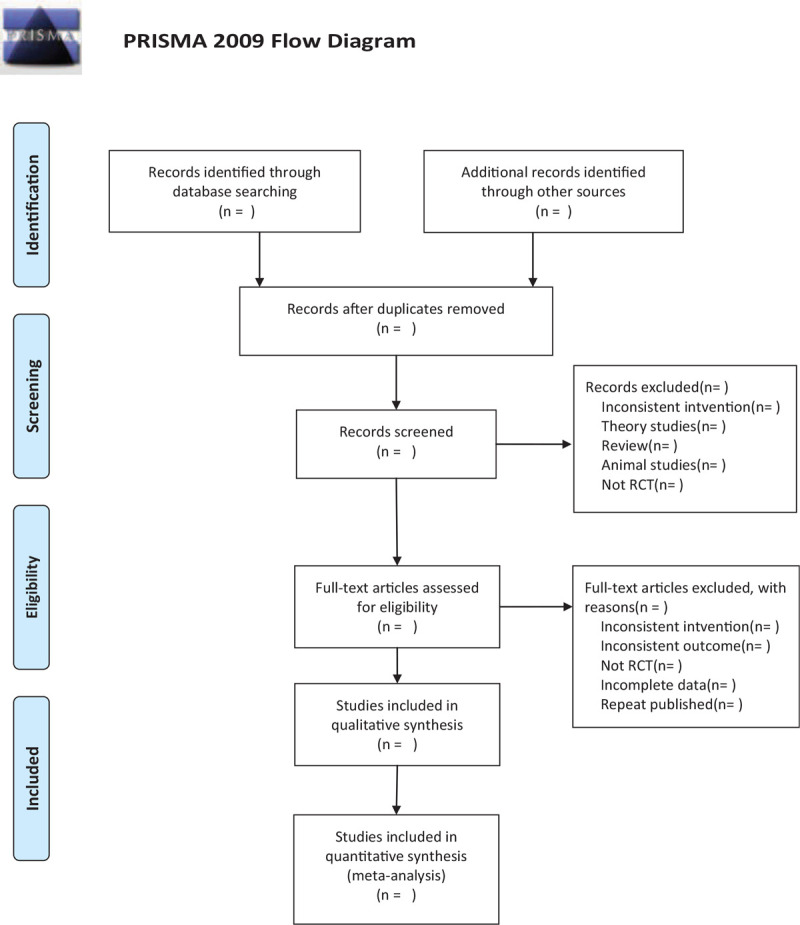
The research flowchart. This figure shows the identification, screening, eligibility and included when we searching articles.

This review will include the following electronic databases from their inception to July 2020: Electronic database includes PubMed, Embase, Cochrane Library, Web of Science, China National Knowledge Infrastructure. (Fig. 1) The research flowchart.

#### 3.6.2 Search strategies

The following MeSH terms and their combinations will be searched:

1. RA;
2. RCT OR RCTs;
3. Zhuang medicine thread moxibustion or Zhuang medicine bamboo cupping therapy or Zhuang medicine fire needle or Zhuang medicine needle pricking therapy or Zhuang medicine hot-sensitive point-probing acupuncture therapy. The search strategy for PubMed is shown in (Table 1). Other electronic data bases will be used the same strategy.

**Table 1:**
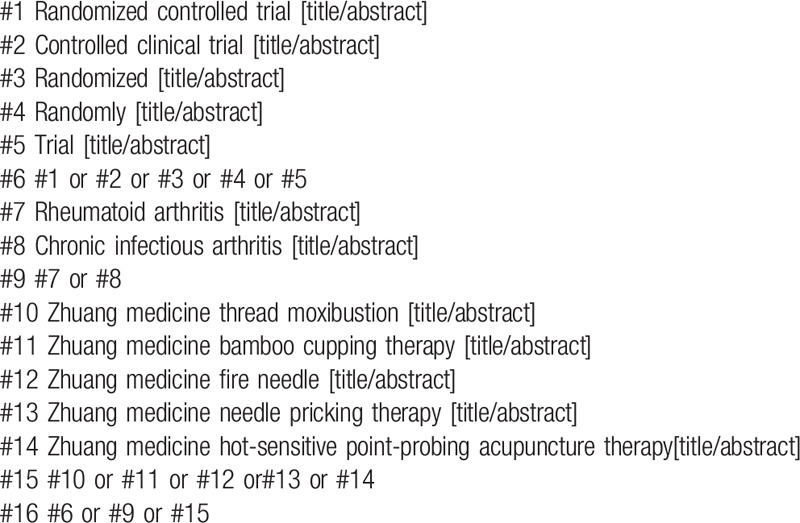
Search strategy in PubMed database.

### 3.7 Data collection and analysis

#### 3.7.1 Studies selection

There will be 2 researchers (YL and DX) carry out the selection of research literature independently using endnote x9 software. We will first make the preliminary selection by screening titles and abstracts. Secondly, we will download full text of the relevant studies for further selection according to the inclusion criteria. If there is any different opinion, 2 researchers will discuss and reach an agreement. If a consensus could not be reached, there will be a third researcher (GF) who make the final decision. The details of selection process will be displayed in the Preferred Reporting Items for Systematic Reviews and Meta-Analyses flow chart.

#### 3.7.2 Assessment of risk of bias

The assessment of risk of bias will be carried out by 2 independent reviewers (YL and WLQ), using the Cochrane Collaboration's “Risk of bias” tool. Study bias will be conducted as either: “unclear,” “low,” or “high” risk for the following criteria: random sequence generation, alloca-tion concealment, blinding, incomplete data, selective outcome reporting, and other bias. The assessment of the bias has caused controversy, there is a need for discussion with a third reviewer (PYZ). The graphic representations of potential bias within and across studies using Rev Man V.5.3.5.

#### 3.7.3 Measures of treatment effect

Statistical analyses will be conducted using the risk ratio with 95% confidence intervals. Odds ratio and relative risk are commonly used for dichotomous outcomes data. For continuous outcomes, the weighted mean difference or the standard mean difference (SMD) will be analyzed.

#### 3.7.4 Unit of analysis issues

The unit of analysis will be the individual participant.

#### 3.7.5 Dealing with missing data

Among the results of several studies with insufficient data or missing data, the corresponding author will be contacted to complement the contents. If the corresponding author cannot be contacted, the data alone will be conducted.

#### 3.7.6 Assessment of heterogeneity

The assessment of heterogeneity will be conducted by Review Manager (V.5.3.5). Chi-squared test and I2value of the forest, plot will be calculated to assess heterogeneity, according to the Cochrane Handbook. The I2value is classified into 4 levels: little or no heterogeneity (0%–40%), moderate heterogeneity (30%–60%), substantial heterogeneity (50%–90%), and considerable heterogeneity (75%–100%).

#### 3.7.7 Assessment of reporting biases

If the numbers of available studies are sufficient, funnel plots will be assessed reporting biases.

#### 3.7.8 Data synthesis

Review Manager (V.5.3.5) will be used to analyze. The test indicated little or no heterogeneity; a fixed effect model will be used for data. The random effect model will be adopted when there is considerable heterogeneity (I2≥50%). If there is considerable variation in results (I2≥75%), the meta-analysis will not be performed. The narrative and qualitative summary will be available.

#### 3.7.9 Subgroup analysis and investigation of heterogeneity

Subgroup analysis will be conducted to assess heterogeneity. The different types of auricular acupuncture (embedding therapy on-ear point, auricular point seeds pressure, bloodletting at auricular points, auricular point on auto chemotherapy) may be affected heterogeneity.

#### 3.7.10 Sensitivity analysis

Sensitivity analysis will be used to assess the robustness of the results. It is possible to determine according to methodological quality, sample size, and analysis-related issues. The studies that follow a sequence will be removed from all the inclusion reviews. The Chi-squared test and I2 value will be used to quantify statistical heterogeneity.

#### 3.7.11 Summary of evidence

The assessment of evidence for all outcomes will be summarized using the Grading of Recommendations Assessment, Development and Evaluation approach. The quality of evidence will be rated as high, moderate, low, and very low quality.

## 4 Discussion

Globally, treatments of RA is a difficult but important problem in the medical field. In Western medicine, treatments of RA emphasize on “target therapy”, with a preference to relieve symptoms. Most patients are treated with non-steroidal anti-inflammatory drugs, DMARDs and biological agents, however, these drugs have serious side effects such as hepatorenal toxicity and immunosuppression, and biological agents are costly so that it could be a financial burden on family.^[9,10]^ Zhuang medicine, as a part of traditional Chinese medicine, has a complete theoretical system and abundant clinical experience. Moreover, various treatments have a remarkable curative effects in RA. According to the theory of Zhuang medicine, there is a unique understanding of RA, which belongs to the category of Fungcaep (a special term used in Zhuang medicine).^[11]^ According to the theory of Zhuang medicine, it is mostly caused by pathogenic toxin, physical weakness, emotional disorder and so on. In fact, the essence of pathogeny is “Shi Du” (dampness toxin). Up to now, the therapies of Zhuang medicine are various, which mainly contains of Zhuang medicine thread moxibustion, Zhuang medicine bamboo cupping therapy, Zhuang medicine fire needle, Zhuang medicine needle pricking therapy, Zhuang medicine hot-sensitive point-probing acupuncture therapy and so on.

The treatment of Zhuang medicine in RA has been widely applied in clinics, which obtains a great effect. It is reported that, for instance, Zhuang medicine cupping combined with pricking blood therapy can improve the clinical symptoms of arthralgia disease patients significantly (Zeng et al 2008).^[12]^ Besides, Zhuang medicine thread Moxibustion can obviously reduce the levels of serum tumor necrosis factor-α (TNF-α) and interleukin-1β (IL-1β) (Xu et al 2014).^[13]^ Through many clinical samples, it is fully realized that Zhuang medicine has its advantages in treating RA, even in curative effects, it is greater than Western medicine, with fewer adverse reactions.

However, the mechanism and standards of treating RA using traditional therapies of Zhuang Medicine are not expounded systematically. In short, this systematic review and meta-analysis can help identify the potential value of Zhuang Medicine in treating RA and improving pain, joint dysfunction and life quality. This study can provide a foundation for the release of RA treatment guidelines and treatment options of RA patients, and thus benefit more patients.

## Author contributions

**Conceptualization:** Yehao Luo, Donghan Xu.

**Data curation:** Donghan Xu, Qiuxia Chen, Fang Gang.

**Data curation:** Yehao Luo.

**Formal analysis:** Yehao Luo, Zhiyong Cao, Lizhen Wang, Donghan Xu.

**Funding acquisition:** Yuzhou Pang, Fang Gang.

**Investigation:** Qiuxia Chen, Lizhen Wang, Donghan Xu.

**Methodology:** Zhiyong Cao.

**Project administration:** Gang Fang, yuzhou pang.

**Quality assessment:** Fang Gang, Yehao Luo.

**Resources:** Donghan Xu, Lizhen Wang.

**Software:** Qiuxia Chen, Donghan Xu, Lizhen Wang, Zhiyong Cao.

**Supervision:** Yehao Luo.

**Validation:** Yehao Luo, Lizhen Wang.

**Writing – original draft:** Yehao Luo, Donghan Xu, Zhiyong Cao, Gang Fang.

**Writing – review & editing:** Donghan Xu, Fang Gang, Yuzhou Pang.
